# The Hospital Saturday Fund Awards

**Published:** 1890-01-25

**Authors:** 


### Fallow Crops.

### THE HOSPITAL SATURDAY FUND AWARDS.

We congratulate the Hospital Saturday Fund authorities that they found themselves with £12,000 to distribute last month in place of the £10,000 at their disposal in 1888. We learn that "the main bases of their grants were efficiency, usefulness, and economy of administration," but before we approve the judgment we should like to know something more of the qualifications of those who appointed themselves to deliver it. There is so great a number of institutions, the circumstances are so various, and the conditions so complex, that we doubt if even the best-informed among us would be quite certain to do justice all round. The more reason, therefore, to hesitate before lightly assuming a function it is next to impossible to perform satisfactorily, and to bestow commendation or, still worse, disapprobation in the wrong place.

When we are dealing with hospitals whose usefulness and efficiency are unquestioned, it would appear only reasonable that the amount of the grant should be governed primarily by the extent of the work performed and the degree in which that work is dependent upon outside help. But if we apply this simple test to the list of awards before us, we are confronted by all kinds of curious anomalies. For instance, the London Hospital, with an average of 644 beds occupied, obtains a grant of £602 11s., while the Brompton Consumption Hospital, with but 297 beds, gets £585 9s., though the latter, we believe, has been of late particularly fortunate in the matter of legacies. Why this difference? Is it to be accounted for by the propensity for appraisement, and can it be contended that the London Hospital is only one-half as efficient, useful, and economical as the other? Again, how is it that Guy's, which has an income from property of £25,000 and has lately made an addition of £100,000 to its capital, is allotted £536 11s. for its 410 beds? These three awards are by far the largest, and serve to illustrate the inconsistencies we have noted.

But the list teems with similar incomprehensibilities, as witness a grant of £273 15s. to the City of London Hospital for Diseases of the Chest with 109 beds, while the Westminster, with 172, only gets £215 2s., or Charing Cross, with 115 beds and a grant of £200 2s., while King's College, with 159, is allotted no more than £186 5s., and University College, with 179, only £181 3s. The Hospital for Sick Children is fortunate in getting £180 14s. with only 78 cots in use, and the West End Hospital, with 10 cots, £72 1s., while the German, with 123 beds, must be content to receive £138 5s., and the National (Queen-square), with 130 beds, £99 12s.

If we compare these awards with those made by the older and surely not less capable body, the Sunday Fund Council, upon the same returns of work, the differences become bewildering. We search vainly for agreement anywhere, and in the case of one small special hospital which obtains a relatively high, if not the very highest, grant from the Saturday Fund, in the returns of the Sunday Fund it does not figure at all.

Who, then, is to decide as to the merits of the several institutions, and is it desirable that an arbitrary power should be vested in an authority constituted like the Board of Delegates of the Hospital Saturday Fund? No doubt the board has done its best according to its lights, but these must be woefully dim or the delegates would not have stumbled so often. Perhaps by next year, if they still intend to venture upon the perilous task of analysing and weighing the respective merits of ninety different and often dissimilar institutions, they will have sought to equip themselves with some of that "diffusive touch" which has been ascribed to Lord Bacon and other judicial masters, else much that is true will still escape them, and as much more that is only error will be taken as truth. The gentlemen who have delivered themselves of judgment upon the hospitals would be sorely put to it, we are afraid, if called upon to describe the processes by which their awards have been arrived at.

Agricola.

